# New Methylene Blue Covalently Functionalized Graphene Oxide Nanocomposite as Interfacial Material for the Electroanalysis of Hydrogen Peroxide

**DOI:** 10.3389/fchem.2021.788804

**Published:** 2021-12-03

**Authors:** Jifang Chen, Ziqing Gao, Ruonan Yang, Huiling Jiang, Lin Bai, Ailong Shao, Hai Wu

**Affiliations:** ^1^ School of Chemistry and Materials Engineering, Fuyang Normal University, Fuyang, China; ^2^ Anhui Province Key Laboratory of Environmental Hormone and Reproduction, Anhui Province Key Laboratory of Embryo Development and Reproductive Regulation, Fuyang, China

**Keywords:** new methylene blue, graphene oxide, covalent immobilization, hydrogen peroxide, electrochemical sensor

## Abstract

New methylene blue (NMB), a phenothiazine dye, was covalently bonded to graphene oxide (GO) using glutaraldehyde as a crosslinking agent, which was characterized by spectroscopic techniques and electrochemistry. The obtained GO–NMB nanocomposite was used as interface material to construct a novel electrochemical sensor for the determination of hydrogen peroxide (H_2_O_2_). The electrochemical sensor based on GO–NMB nanocomposite exhibited excellent electrocatalytic activity for the reduction of hydrogen peroxide (H_2_O_2_), which was also enhanced by GO within the GO–NMB nanocomposite. With the optimized experimental conditions, the developed sensor showed high sensitivity (79.4 μA mM^−1^ cm^−2^) for electrocatalytic determination of H_2_O_2_ at the applied potential of −0.50 V in the concentration range of 0.000333 to 2.28 mΜ. The low limit of detection (1.35 μM), good reproducibility, and high stability of the sensor suggests that the electrochemical sensor based on the GO–NMB nanocomposite possesses obvious advantages, which paves a new avenue to functionalize GO for obtaining electrode interface materials.

## Introduction

Electrochemical sensors are powerful and attractive analytical tools due to their advantages, such as high sensitivity, accurate signal conversion, fast response, low manufacturing cost, easy miniaturization, and integration. These performances, however, are influenced, to some extent, by electrode substrates and interfacial materials (Masibi et al., 2018; Asadian et al., 2019; Chinnadayyala and Cho, 2020). Therefore, a diversity of novel nanomaterials with well-controlled physicochemical features, electrocatalytic activities, shape, and dimension have been produced and used as interfacial materials owing to the high reactive surfaces area and small particle size (Burda et al., 2005). Recently, carbon-based nanomaterials, such as graphene oxide, carbon nanotubes, mesoporous carbon, and fullerenes, have been applied to the fabrication of electrochemical biosensors due to their high electrical conductivity, large specific surface area, and easy modification (Wu et al., 2014a; Asadian et al., 2019; Alam et al., 2020).

Graphene oxide, a “rising-star” carbon material, is considered an ideal candidate due to the presence of oxygen-containing functional groups including hydroxyl, carboxyl, and epoxy groups (Ramesh et al., 2004), which provides reactive sites for covalent or noncovalent functionalization with a wide variety of catalysts (Nanda et al., 2015; Georgakilas et al., 2016), metallic nanoparticles (Tucek et al., 2016; Liu et al., 2018), and electron mediators (Subbiah et al., 2017; Xue et al., 2018). Our group has conveniently synthesized a zinc porphyrin dye (YD) noncovalently functionalized graphene oxide (GO) nanohybrid (GO@YD) through hydrogen bonding and π–π stacking interactions. The GO@YD nanohybrid-based electrochemical sensor showed high sensitivity and selectivity for AA detection (Wu et al., 2018). However, due to the relatively weak non-covalent interaction, there is poor dispersion of non-covalently functionalized carbon nanomaterials in aqueous solution, posing a serious challenge for practical applications (Guo et al., 2019; Park et al., 2021). Covalent functionalization of GO with the anchored molecules by edge-group modification and basal plane modification can improve its structural stability, dispersion, electrical conductivity, and potential catalytic activity (Park et al., 2021). Guo et al. first used thiolated graphene oxide (GO–O–SH) as a substrate to covalently link gold nanorods (AuNRs) on the surface of GO, obtaining the GO@AuNRs composites for an ultra-sensitive Raman probe (Guo et al., 2019). Kong et al. reported the covalent functionalization of reduced graphene oxide aerogel (RGOA) by diazotization and amidation to graft conducting polymer polyaniline (PANI) onto the surface of RGOA. The covalent bonding between RGOA and polyaniline not only enhances the electrical conductivity but also prevents the agglomeration of graphene nanosheets (Li et al., 2019). More recently, Xia et al. (2021) constructed a bioelectrochemical sensor based on covalent immobilization bionic oxidase iron porphyrin with chloroprene-functionalized graphene oxide surface for sensitive detection of catechol. Obviously, the covalent functionalization of graphene oxide constitutes a subject of interest for enhancing the catalytic sensitivity of electrochemical sensors.

A series of organic dyes or compounds including methylene blue, Prussian, neutral red, ferrocene, and covalent organic frameworks have been modified on carbon nanostructures for constructing electrochemical sensors, which showed good electrocatalytic properties in determination of different analytes. New methylene blue (NMB), a phenothiazine dye, is a good electron transfer mediator due to its conjugated planar structure (Liu et al., 1996). Its special chemical structure with imino groups on the heteroaromatic ring makes it easier to be functioned with other materials. We have covalently immobilized NMB and horseradish peroxidase-labeled penicillin polyclonal antibody (HRP-PePAb) on a glassy carbon electrode (GCE) to prepare an amperometric immunosensor for sensitive determination of penicillin in milk (Wu et al., 2014b). Zhu et al. reported the electrochemical properties of NMB/silicon oxide nanocomposite mediator and its application in hydrogen peroxide biosensor (Yao et al., 2005); however, the direct current response of NMB alone is weak, affecting the accuracy of detection. Herein, NMB was used as a mediator and was covalently immobilized on GO to obtain the GO–NMB nanocomposite. A novel electrochemical sensor based on the GO–NMB nanocomposite was proposed for the sensitive determination of hydrogen peroxide in low concentration, which showed excellent detection performances.

## Experimental

### Chemicals and reagents

Glutaraldehyde (25 wt% in H_2_O) was bought from Sinopharm Chemical Reagent Co., Ltd. (Shanghai, China). Graphene oxide (GO) was purchased from XFNANO Materials Co. Ltd. (Nanjing, China). New methylene blue (NMB) and hydrogen peroxide (H_2_O_2_, 30%) were purchased from J&K Scientific (China). The 0.1 M phosphate buffer solution (PBS, pH 7.0) with 0.1 M KCl was prepared with KH_2_PO_4_, Na_2_HPO_4_·12H_2_O, and KCl solutions, and its pH was adjusted by NaOH and HCl solutions with a pH meter (Mettler Toledo). Aqueous solutions were prepared with high purity water.

### Covalent immobilization of grapheme oxide and new methylene blue

**SCHEME 1:**
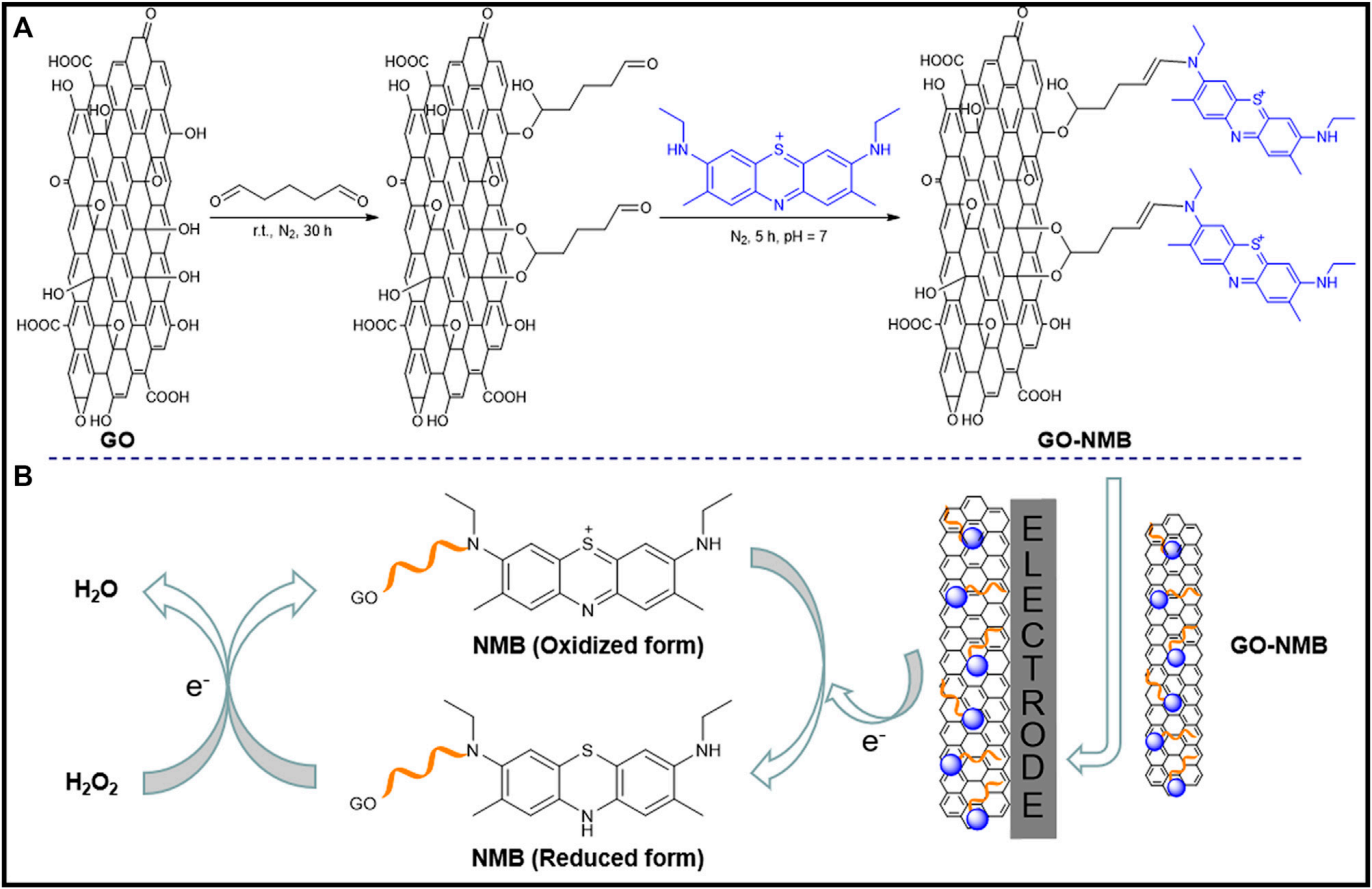
Electrocatalytic mechanism of new methylene blue (NMB) for the reduction of hydrogen peroxide (H_2_O_2)_.

First, 5 mg of GO was dispersed in 5 ml of 5% glutaraldehyde and stirred for 12 h at nitrogen atmosphere. The pH of the solution was adjusted to 7.0 with 10% sodium hydroxide solution, and 5 ml of 0.4 mM NMB solution was then added drop by drop with stirring for 5 h. The mixed solution was dialyzed at room temperature for 30 h until the high-purity water was colorless and was then centrifuged at 7,500 rpm/ s for 20 min. The precipitate was dried at 55°C in a vacuum drying oven to obtain the GO–NMB nanocomposite. The cross-linking mechanism was based on the acetal or hemiacetal reaction between hydroxyl groups of the GO sheets and aldehyde on glutaraldehyde (Podsiadlo et al., 2007; Hu et al., 2011), and the aldimine condensation between the aldehyde group at the other end of glutaraldehyde and the imine of NMB. The covalent bonding processes are illustrated in Scheme 1A.

### Fabrication of the modified electrodes

Prior to the modification, GCE was sequentially polished to a mirror surface with 0.3 and 0.05 µm of alumina slurry and was then rinsed with ultrapure water. After continuous sonication in nitric acid (*v/v*, 1:1), anhydrous ethanol, and purified water for 30 s, respectively, GCE was dried with nitrogen gas. To obtain a homogeneous suspension, 8 mg of the prepared GO–NMB nanocomposite was dissolved in 1.0 ml of ultrapure water and sonicated. Then 5 µl of homogeneous dispersion solution of GO–NMB was dropped onto the surface of cleaned GCE and dried at ambient temperature. For comparison, NMB/GCE and GO/GCE were prepared using NMB and GO solution with the same condition, respectively. The construction process is shown in Scheme 1B.

### Apparatus and measurements

Spectral characterization of GO, NMB, and GO@NMB composites were performed using UV-vis spectrometer (PerkinElmer 750s), fluorescence spectrometer (HITACHI F-7000), and FT-IR spectrometer (Thermo Fisher Nicolet iS50). Electrochemical measurements were performed with a CHI660E electrochemical workstation (Chenhua, Shanghai) using a conventional three-electrode system. A modified GCE, an Ag/AgCl electrode (3 M KCl), and a platinum wire were used as the working electrode, reference electrode, and auxiliary electrode, respectively. Before every electrochemical experiment, the prepared GO-NMB/GCE was first scanned in the potential range from 0.1 to −1.2 V for 10 cycles using cyclic voltammetry, obtaining stable redox current. Cyclic voltammetry was performed in the potential range of −0.6 to 0.6 V with a scan rate of 100 mV s^−1^.

## Results and discussion

### Characterization of the graphene oxide–new methylene blue nanocomposite

**FIGURE 1:**
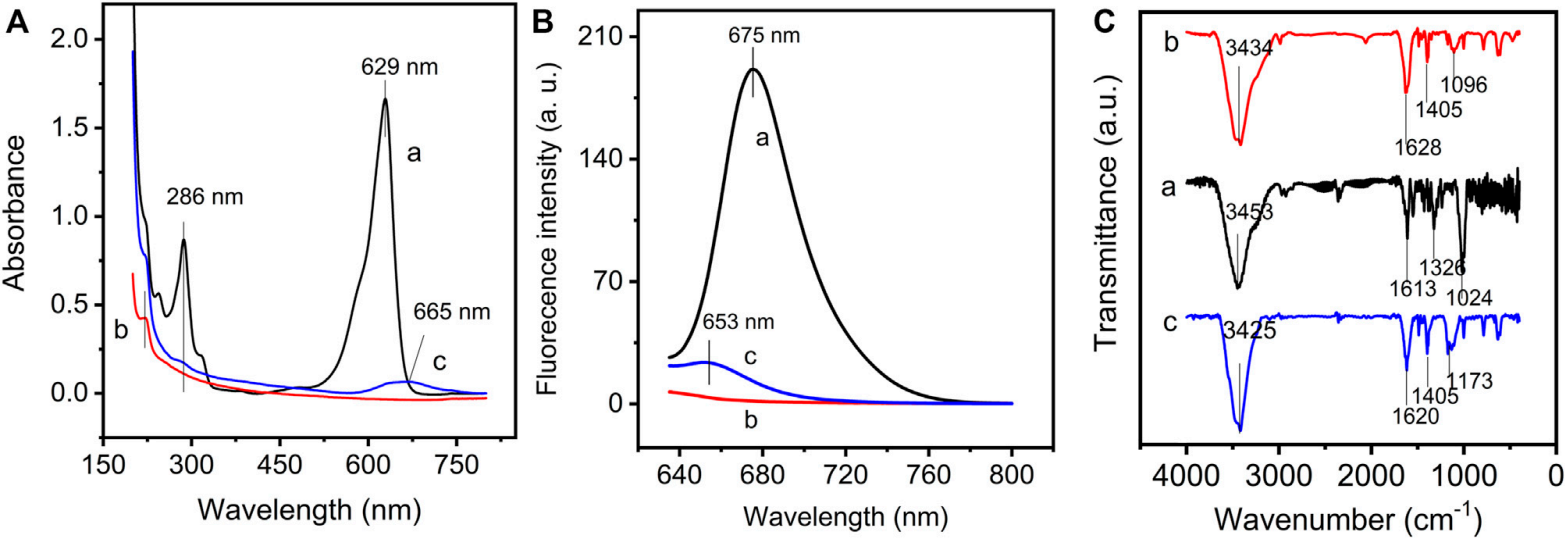
**(A)** UV–vis spectra, **(B)** fluorescence spectra, and **(C)** FTIR spectra of new methylene blue (NMB) (curve a), graphene oxide (GO) (curve b), and graphene oxide–new methylene blue (GO–NMB) (curve c).

The covalent bonding reaction between GO and NMB was successfully confirmed by UV-Vis, fluorescence, and FT-IR spectroscopy. In Figure 1A, NMB displays a strong absorbance at 629 nm corresponding to the π–π* transition of NMB aromatic chromophore (curve a) (Paul et al., 2017, 2020). After covalently bonding with GO (curve c), this prominent absorption shifts from 629 to 665 nm, which suggest that the plane of NMB is close to the GO plane and causes π–π stacking interaction between the two conjugate planes. The result is also proven by the following structural optimization. The absorption at 286 nm attributed to π–π* transition, which also occurred on the GO–NMB nanocomposite without a significant shift in wavelength, which indicates that the structure of bonded NMB on GO was not affected by covalent bonding reaction. Furthermore, both GO and GO–NMB show a weak absorption peak at about 221 nm, which is ascribed to π–π* transition of C–C and C = C bonds in the sp^2^ hybrid regions of GO (curve b) (Cuong et al., 2010). Therefore, the results demonstrate that the GO–NMB nanocomposite remains the main structure and properties of the GO and NMB monomer.

The emission spectra of NMB, GO, and GO-NMB were recorded to study the interaction between GO and NMB. NMB has a maximum emission peak at 675 nm but GO has no fluorescence when excited at 629 nm (Figure 1B). After the interaction with GO, the fluorescence of NMB was obviously quenched by GO with a blue shift from 675 to 653 nm, mirroring the bathochromic shift that occurred in the UV-vis spectrum of GO-NMB (Huang et al., 2019).

The FT-IR spectrum of GO, NMB, and GO–NMB are presented in Figure 1C. NMB shows a characteristic peak at 3,453 and 1,613 cm^−1^ (curve a), which are the stretching vibration of N–H and the ring extension of the benzene ring, respectively. The symmetric C–N extension and C–C extension vibration can be observed at 1,326 and 1,024 cm^−1^, respectively (Ovchinnikov et al., 2007; Sharma et al., 2013; Zhang et al., 2016). For GO (curve b), the vibrational peaks at 3,434, 1,628, 1,405, and 1,096 cm^−1^ can be assigned to O–H stretching, sp^2^-hybridized C=C groups stretching, O–H bending vibrations, and C–O stretching of epoxy or alkoxy groups, respectively (Basirun et al., 2013; Golsheikh et al., 2013; Zhang et al., 2016). The spectrum of GO–NMB (curve c) showed a redshift from 1,613 to 1,620 cm^−1^, probably due to an increase in its conjugation after bonding of NMB. Noteworthy, the decrease in C–O vibration of epoxy or alkoxy bonds at 1,096 cm^−1^ and the appearance of the band at 1,173 cm^−1^ assigned to C–N indicate the reaction between the –NH of NMB (Hu et al., 2013; Chen et al., 2018), but the band of O–H at 1,405 cm^−1^ did not decrease obviously, which shows that small bonded NMB molecule did not affect the main structure of GO.

### Electrochemical behavior of graphene oxide-new methylene blue/glassy carbon electrode

**FIGURE 2:**
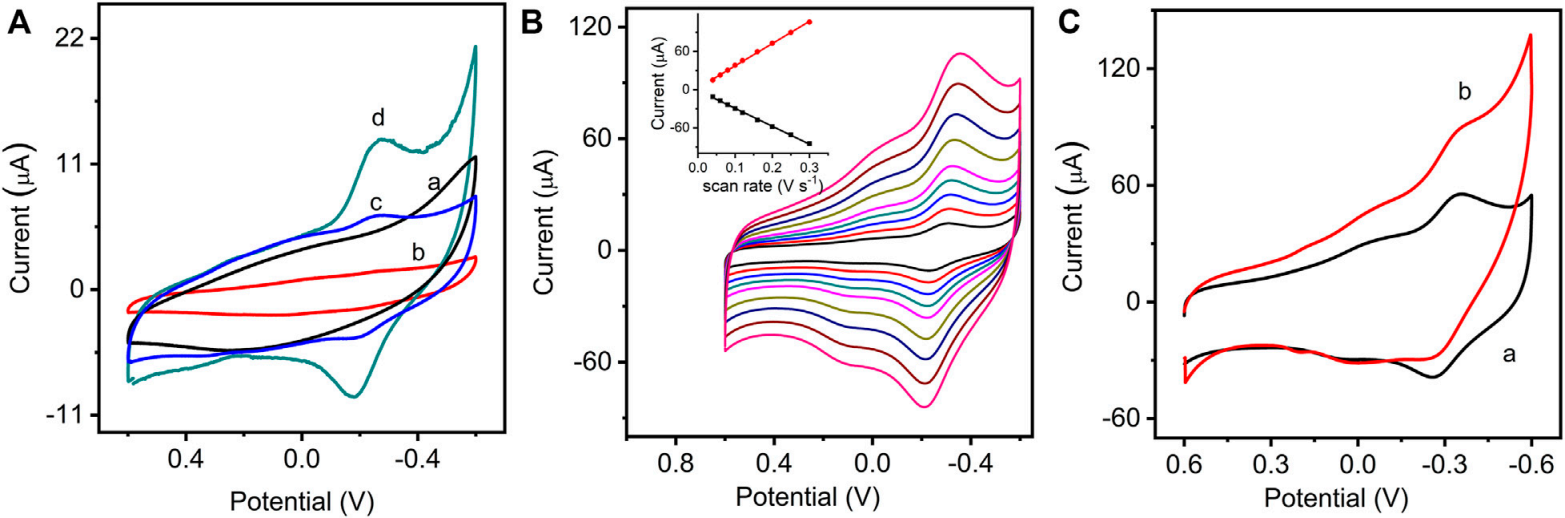
**(A)** Cyclic voltammograms (CVs) of GO/glassy carbon electrode (GCE) (a), NMB/GCE (b), GO/NMB/GCE (GO and NMB physical mixture) (c), and GO–NMB/GCE (d) in 0.1 M phosphate buffer solution (PBS) (pH 7.0, 0.10 V s^−1^). **(B)** CVs of GO–NMB/GCE at different scan rates of 0.04, 0.06, 0.08, 0.10, 0.12, 0.16, 0.20, 0.25, and 0.30 V s^−1^ (inset: the relationship between the redox peak current and the square root of the scan rate). **(C)** CVs of GO–NMB/GCE in 0.1 M PBS in the absence (curve a) and presence (curve b) of hydrogen peroxide (H_2_O_2_).

The electrochemical performance of the modified electrodes was evaluated by cyclic voltammetry. Figure 2A shows cyclic voltammograms (CVs) of different modified electrodes in 0.1 M PBS. It can be found that the GO/GCE (curve a) and NMB/GCE (curve b) have no significant redox peaks in the scanned potential range. A pair of redox peaks can be found on the GO/NMB/GCE (curve c), which is due to the physical adsorption of NMB on GO. By comparison, GO–NMB/GCE exhibited a higher redox peak current and a more reversible redox process (curve d). Figure 2B presents the CVs of GO-NMB/GCE in 0.1 M PBS with increasing scan rates from 0.04 to 0.3 V s^−1^. The redox peak currents increased linearly with the scan rates (inset in Figure 2B), indicating a surface-controlled redox process. The prepared electrochemical sensor based on the synergistic effect of GO–NMB nanocomposite showed good electrocatalytic activity for the reduction of H_2_O_2_. As shown in Figure 2C, in the presence of H_2_O_2_, the reduction peak current of NMB on GO–NMB increased along with the decrease in the oxidation peak current, showing a typical electrocatalytic process. All the results indicate that covalently bonded NMB on GO–NMB nanocomposite can be used as an excellent electron mediator for electrocatalytic determination of H_2_O_2_. Moreover, the electron transfer rate of NMB can be promoted by GO, and the analytical sensitivity can be enhanced with their synergistic effect.

### Optimization of experimental conditions

**FIGURE 3:**
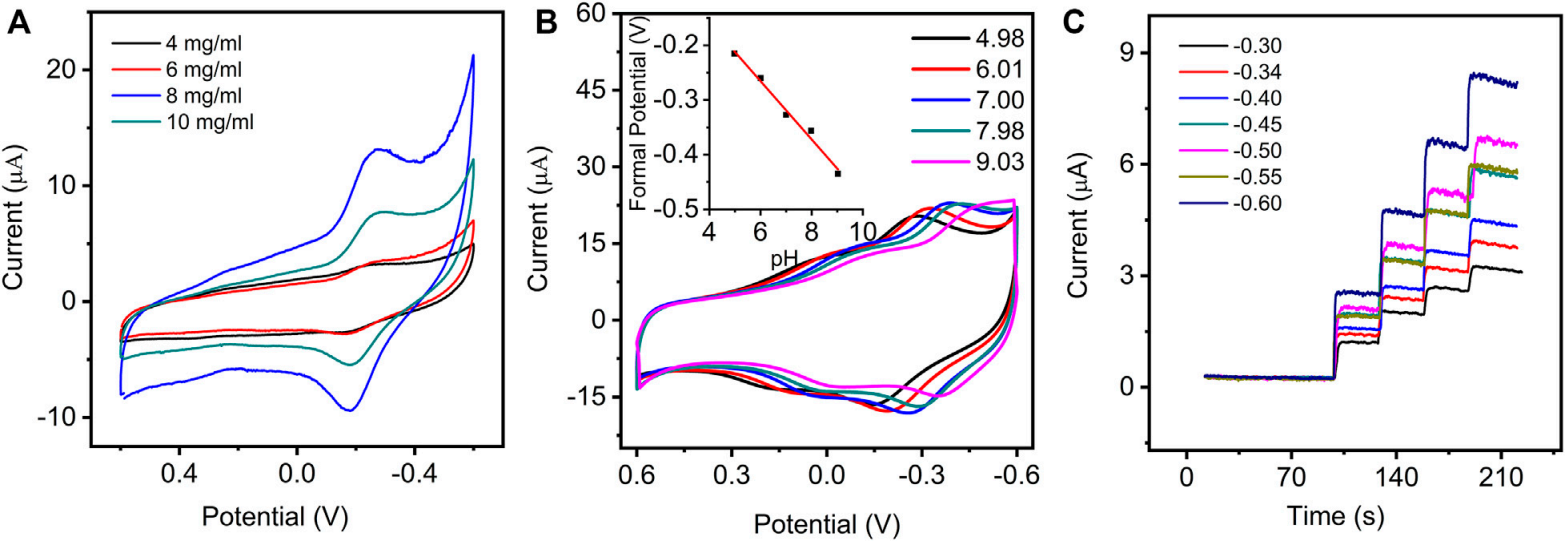
**(A)** CVs of GO–NMB/GCE in 0.1 M PBS (pH 7.0) with various concentrations of GO–NMB modified solution from 4 to 10 mg ml^−1^. **(B)** CVs of GO–NMB/GCE in PBS with various pH (inset: the plot of the relationship between formal potential and different pH). **(C)** The amperometric current−time curves of GO–NMB/GCE at different applied potential with successive injections of the same volume of hydrogen peroxide (H_2_O_2_).

The experimental conditions, including the modified concentration of GO–NMB, pH of PBS, and applied potential for detecting H_2_O_2_, were optimized to achieve the best electrochemical response. As shown in Figure 3A, the highest peak currents of GO–NMB/GCE were obtained when the GO–NMB concentration was up to 8 mg ml^−1^. The current of the electrode decreased significantly at the GO–NMB concentration of 10 mg/ ml, which suggested that the thickness of the modified layer would affect the electron transfer process.

The influence of pH on the electrochemical behavior of the GO–NMB/GCE in 0.1 M PBS was investigated in the range of 4.98 to 9.03 (Figure 3B). A liner regression equation was obtained from a plot of formal potential with pH: *E* ^θ^ (V) = −0.0542–0.0534 pH (R = 0.9932). The slope (−53.4 mV pH^−1^) is very close to the theoretical value of 59 mV pH^−1^, indicating that equal proton and electron are involved in the redox process (Li et al., 2009). On the basis of the above results and a previous study, a possible mechanism for this electrochemical process of GO–NMB on the electrode is described in Scheme 1B (Mollarasouli et al., 2017). The peak currents increased gradually until they reached the maximum at pH 7.00 (Figure 3C); therefore, pH 7.00 for 0.1 M PBS was chosen for further experiments.

In addition, the applied potentials for amperometric determination of H_2_O_2_ were optimized. As shown in Figure 3D, the catalytic current responses toward H_2_O_2_ exhibit excellent and quick amperometric responses at all the applied potentials, but the catalytic sensitivity toward the same concentration of H_2_O_2_ gradually increases as the potential is up to −0.50 V, whereas the response decreases at −0.55 V and then increases again at −0.60 V, which is consistent with the CV response. Considering that more negative potential will cause greater interference, −0.50 V was selected as the optimum applied potentials to determine H_2_O_2_.

### Electrocatalytic analysis of presented sensor

**FIGURE 4:**
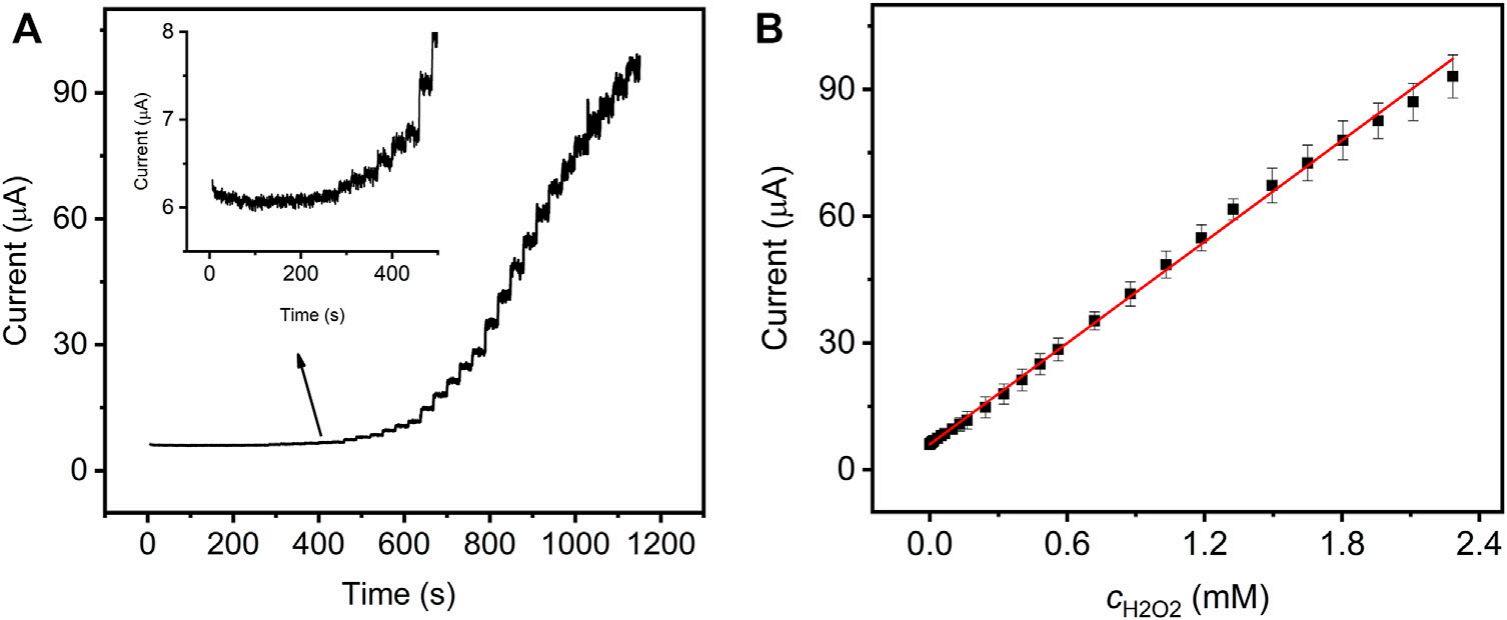
**(A)** Amperometric response of GO–NMB/GCE with successive additions of H_2_O_2_ in 0.1 M PBS (pH 7.0) at the applied potential of −0.50 V. **(B)** Corresponding calibration plots between catalytic currents and H_2_O_2_ concentrations.

To evaluate the electrocatalytic activity of GO–NMB/GCE, H_2_O_2_ was selected as the analyte due to its extensive application in medicine, food products, and biosensor developments (Fan et al., 2016). Figure 4A shows the typical amperometric response of the presented sensor with successive injections of H_2_O_2_ to constantly stir 0.1 M PBS. The current response grew linearly with the concentration of H_2_O_2_ in the range of 0.00033 to 2.28 mM (Figure 4B). The response sensitivity is up to 79.4 μA mM^−1^ cm^−2^, and the limit of detection (LOD) was calculated to be 1.35 μM (Lin et al., 2012). The analytical performances of the sensor and reported sensors in terms of linear range, sensitivity, and LOD were compared and are summarized in Table 1. The presented sensor shows a lower LOD and higher sensitivity, which indicated that the GO–NMB could be an alternative material for the construction of H_2_O_2_ electrochemical sensor. However, it must be noted that the relatively narrow linear detection range may be due to the low amount of NMB covalently bonded on the surface of GO, which will be further optimized in our later research.

**TABLE 1:** Comparison of the presented sensor for electrochemical determination of hydrogen peroxide (H_2_O_2_) with other reported sensors.

Sensors^a^	Linear range (µM)	LOD (µM)	Sensitivity (µA/mM/cm^2^)	Ref
NiO–NSs/CF-1801/GCE	200–3750	0.01303	23.30	Liu et al. (2021)
PB/MoS_2_–rGO/GCE	0.3–1,150	0.14	−	Cheng et al. (2017)
MWCNTs50/PMB/CC	100–3,000	21.7	108	Peña et al. (2011)
B-doped dGN/GCE	5–1,955	0.48	492.71	Hsu et al. (2021)
Hb/3.0 PAMAM–AuNPs/GCE	20–950	6.1	35.07	Elancheziyan and Senthilkumar, (2019)
AuNPs–PB–GO/GCE	3.8–5,400	1.3	−	Liu et al. (2021)
AuNPs–GO/GCE	50–4,600	25	−	Bas, (2015)
GQDs–CS/MB/GC	1–2,900	0.7	−	Mollarasouli, et al. (2017)
	2,900–11,780			
GO@NMB/GCE	0.33–2,280	1.35	79.4	This work

Note. a NiO–NSs, Nickel oxide nanosheets; CF-1801, three-dimensional (3D) carbon foam (CF) network; PB, Prussian blue; MWCNTs50/PMB, multiwalled carbon nanotubes (MWCNT) into a poly(-methylene blue) (PMB) film; Hb/3.0PAMAM–AuNPs, hemoglobin (Hb) was covalently immobilized on polyamidoamine (PAMAM) dendrimer encapsulated with gold nanoparticles; (AuNPs); GQDs-CS, graphene quantum dots (GQDs) functionalized with chitosan; MB, methylene blue; LOD, limit of detection.

### Properties and application of the presented sensor

**FIGURE 5:**
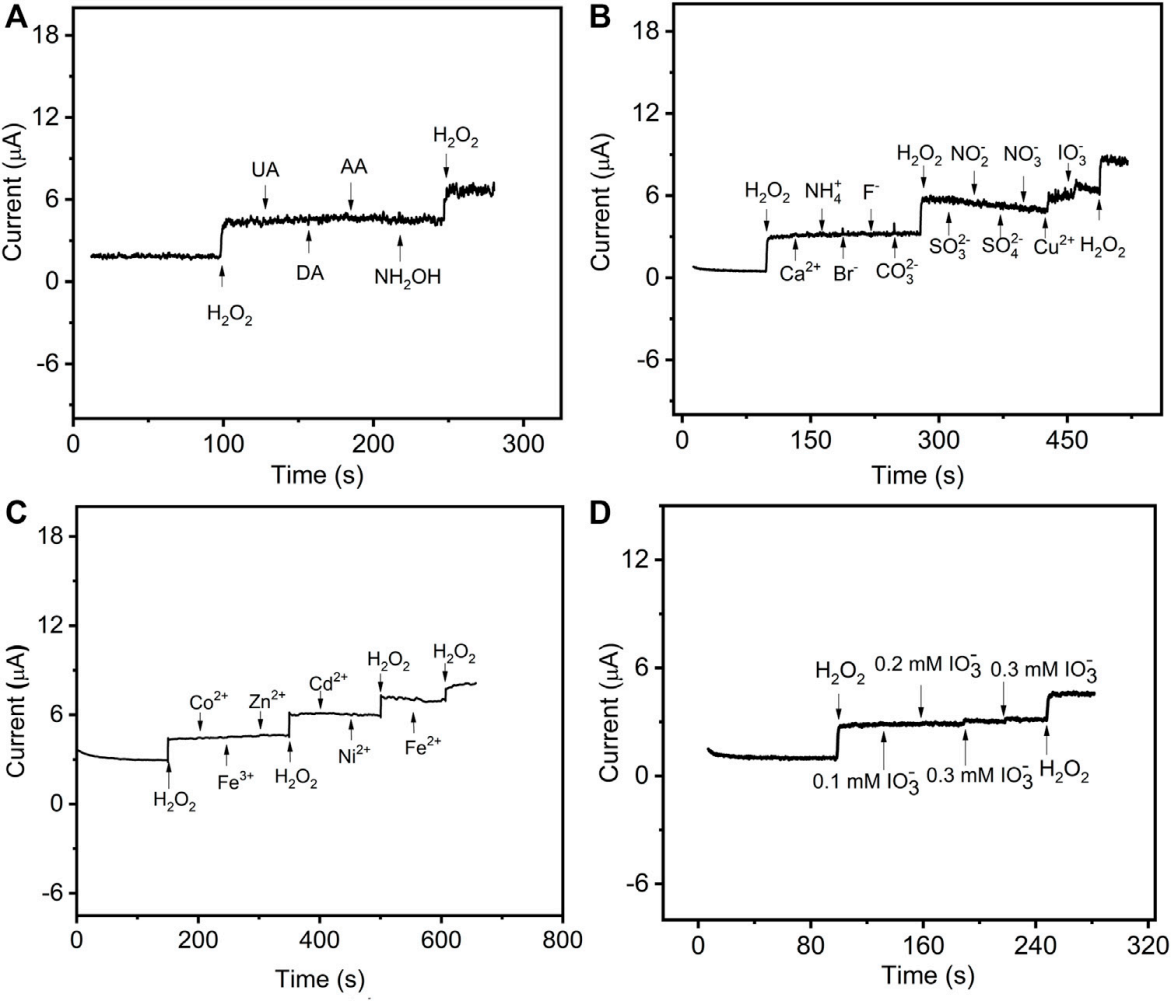
The current–time curve of GO–NMB/GCE in 0.1 M stirring PBS with successive injections of **(A)** 0.05 mM H_2_O_2_, 0.3 mM uric acid (UA), dopamine (DA), ascorbic acid (AA), hydroxylamine hydrochloride (NH_2_OH), and 0.05 mM H_2_O_2_. **(B)** 0.05 mM H_2_O_2_, 0.3 mM Ca^2+^, NH_4_^+^, Br^−^, F^−^, CO_3_^2−^, SO_3_^2−^, NO_2_^−^, SO_4_^2−^, NO_3_^−^, Cu^2+^, IO_3_^−^, and 0.05 mM H_2_O_2_ **(C)** 0.05 mM H_2_O_2_ and various concentrations of Cu^2+^. **(D)** 0.05 mM H_2_O_2_ and various concentrations of IO_3_^−^.

The analytical properties of the presented sensor, including selectivity, stability, and reproducibility, were evaluated by sensing H_2_O_2_. The amperometric responses of the sensor were performed with successive additions of 0.05 mΜ H_2_O_2_ and 0.3 mM interfering substances, including uric acid (UA), dopamine (DA), ascorbic acid (AA), hydroxylamine hydrochloride (NH_2_OH), Co^2+^, Fe^3+^, Zn^2+^, Cd^2+^, Ni^2+^, Fe^2+^, Ca^2+^, Ag^+^, Hg^2+^, NH_4_^+^, Cu^2+^, Br^−^, F^−^, CO_3_^2−^, SO_3_^2−^, NO_2_^−^, SO_4_^2−^, NO_3_^−^, and IO_3_^−^ (Figures 5A−C). The current responses on H_2_O_2_ were not interfered by the injection of other substances except for 0.3 mM IO_3_^−^, Cu^2+^, Ag^+^, and Hg^2+^. As shown in Figure 5D, the interference of IO_3_^−^ can be avoided when its concentration is lower than 0.2 mM. Similarly, as the concentration of Cu^2+^ and Ag^+^ decreased to 0.2 and 0.15 mM, respectively (Supplementary Figure S1), the interfering current can be negligible. However, the interfering current is neglectable when the concentration of Hg^2+^ is as low as 0.03 mM. Therefore, it was concluded that the prepared GO–NMB/GCE sensor presented a good selectivity for the determination of H_2_O_2_ except for the presence of Hg^2+^ and high concentration of IO_3_^−^, Cu^2+^, and Ag^+^.

**FIGURE 6:**
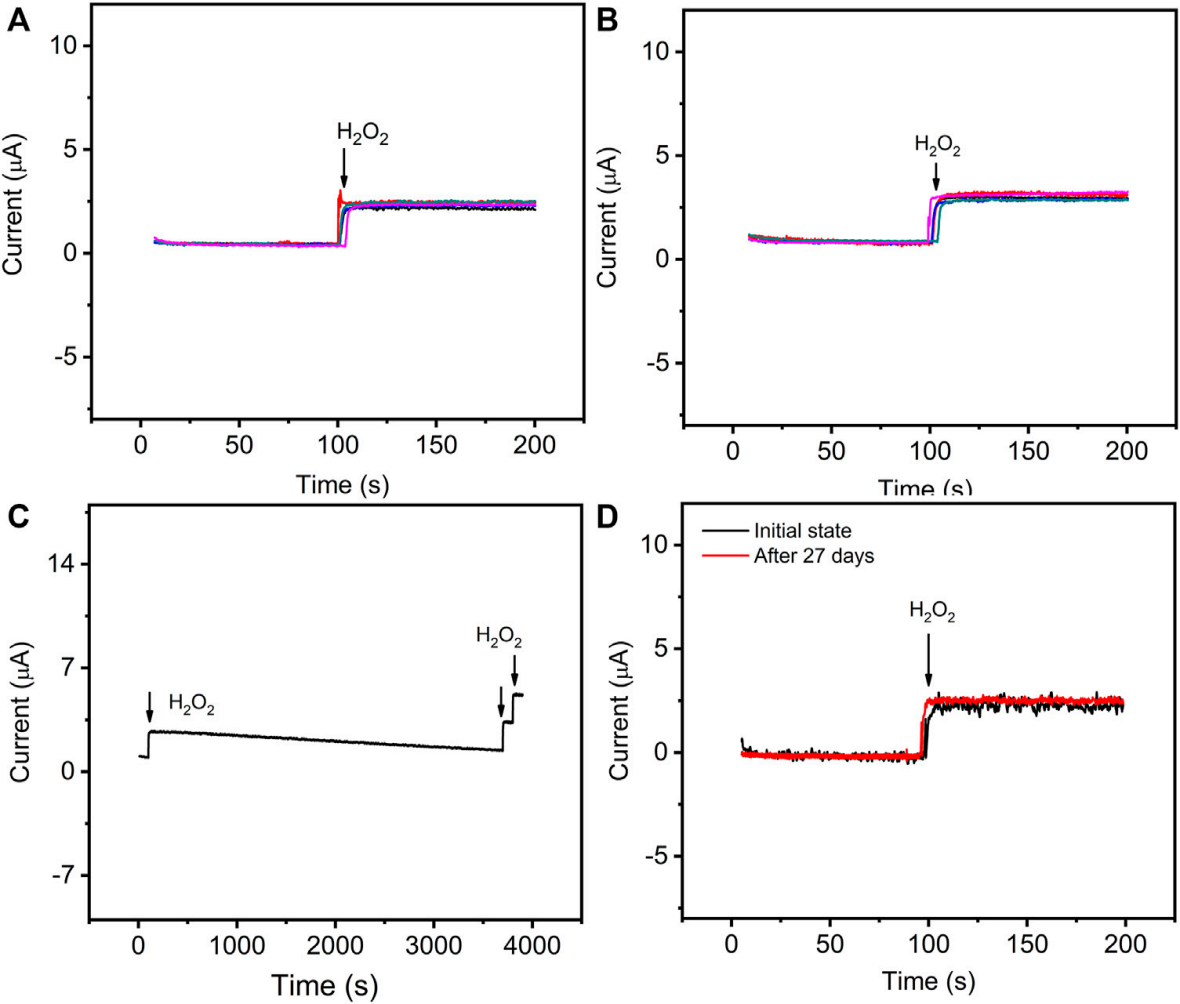
The current–time curves of GO–NMB/GCE toward 83 μM H_2_O_2_ prepared with the same electrode for five times **(A)** and five different electrodes **(B)**. **(C)** Successive current response of the GO–NMB/GCE toward 83 μM H_2_O_2_ in 0.1 M PBS before and after 1-h response. **(D)** Long-term storage stability of the sensor for the response toward 83 μM H_2_O_2_ before and after 27 days.

Additionally, the reproducibility of GO–NMB/GCE was investigated under the same experimental conditions in the section above (Figures 6A, B). The intra-reproducibility of the same modified GCE was carried out by comparing the determination of the same H_2_O_2_ concentration for five times. The relative standard deviation (RSD) is 6.72%. Moreover, RSD was 4.98% for five independently prepared sensors toward H_2_O_2_, indicating a good inter-reproducibility. Therefore, the proposed sensor presents excellent reproducibility for reliable analysis.

The operation of the sensor was studied by continuous response on 83 μM H_2_O_2_ for 1 h. As shown in Figure 6C, the catalytic current remains relatively stable, and moreover, it still shows a sensitive response toward H_2_O_2_ after continuous use for 1 h. The long-term storage stability was examined by comparing the initial response current and the catalytic current after 27 days of storage toward the same concentration of H_2_O_2_ (Figure 6D). Obviously, no significant change in the catalytic current was observed. With the excellent stability, the sensor could serve as a practical electrochemical sensor for H_2_O_2_ detection.

To validate the reliability of the presented senor, diluted disinfector samples have been detected and evaluated by the standard addition method and traditional potassium permanganate method. The acceptable recoveries from 92.4% to 95.8% have been obtained; moreover, the determined results are consistent with those of the potassium permanganate method.

## Conclusion

In conclusion, NMB was successfully covalently immobilized onto GO, which was confirmed by spectroscopic and electrochemical methods. Based on the GO–NMB nanocomposite, a novel electrochemical sensor was proposed for the application in H_2_O_2_ determination. The results from the electrochemical characterization of the sensor suggested that covalent bonded NMB on GO–NMB nanocomposite could be used as an excellent electron mediator for electrocatalytic reduction of H_2_O_2_. Moreover, GO could promote the electron transfer rate of NMB and enhance the response sensitivity. The electrochemical properties of GO–NMB nanocomposite were better than that of the physical mixture of GO and NMB modified electrode. With the synergistic effect, the sensor showed the advantages of high sensitivity, good stability, and reproducibility, and low limit of detection. With these advantages, the method provides a new avenue for the construction of electrochemical sensors based on the covalent functionalized GO composites.

## Data Availability

The original contributions presented in the study are included in the article/Supplementary Material. Further inquiries can be directed to the corresponding authors.

## References

[B1] Alam M. S. Bishop B. Chen N. Safari S. Warter V. Byrne J. M. (2020). Reusable Magnetite Nanoparticles-Biochar Composites for the Efficient Removal of Chromate from Water. Sci. Rep. 10 (1), 19007–19018. 10.1038/s41598-020-75924-7 33149170 PMC7642354

[B2] Asadian E. Ghalkhani M. Shahrokhian S. (2019). Electrochemical Sensing Based on Carbon Nanoparticles: A Review. Sensors Actuators B: Chem. 293, 183–209. 10.1016/j.snb.2019.04.075

[B3] Bas S. Z. (2015). Gold Nanoparticle Functionalized Graphene Oxide Modified Platinum Electrode for Hydrogen Peroxide and Glucose Sensing. Mater. Lett. 150, 20–23. 10.1016/j.matlet.2015.02.130

[B4] Basirun W. J. Sookhakian M. Baradaran S. Mahmoudian M. R. Ebadi M. (2013). Solid-phase Electrochemical Reduction of Graphene Oxide Films in Alkaline Solution. Nanoscale Res. Lett. 8, 397–405. 10.1186/1556-276X-8-397 24059434 PMC4015787

[B5] Burda C. Chen X. Narayanan R. El-Sayed M. A. (2005). Chemistry and Properties of Nanocrystals of Different Shapes. Chem. Rev. 105 (4), 1025–1102. 10.1021/cr030063a 15826010

[B6] Chen C. Zhu X. Chen B. (2018). Covalently Cross-Linked Graphene Oxide Aerogel with Stable Structure for High-Efficiency Water Purification. Chem. Eng. J. 354, 896–904. 10.1016/j.cej.2018.08.034

[B7] Cheng Z. Shen Q. Yu H. Han D. Zhong F. Yang Y. (2017). Non-enzymatic Sensing of Hydrogen Peroxide Using a Glassy Carbon Electrode Modified with the Layered MoS2-Reduced Graphene Oxide and Prussian Blue. Microchim. Acta 184 (12), 4587–4595. 10.1007/s00604-017-2503-x

[B8] Chinnadayyala S. R. Cho S. (2020). Porous Platinum Black-Coated Minimally Invasive Microneedles for Non-enzymatic Continuous Glucose Monitoring in Interstitial Fluid. Nanomaterials 11 (1), 37–51. 10.3390/nano11010037 PMC7824010 33375593

[B9] Cuong T. V. Pham V. H. Tran Q. T. Hahn S. H. Chung J. S. Shin E. W. (2010). Photoluminescence and Raman Studies of Graphene Thin Films Prepared by Reduction of Graphene Oxide. Mater. Lett. 64 (3), 399–401. 10.1016/j.matlet.2009.11.029

[B10] Elancheziyan M. Senthilkumar S. (2019). Covalent Immobilization and Enhanced Electrical Wiring of Hemoglobin Using Gold Nanoparticles Encapsulated PAMAM Dendrimer for Electrochemical Sensing of Hydrogen Peroxide. Appl. Surf. Sci. 495, 143540–143547. 10.1016/j.apsusc.2019.143540

[B11] Fan S. Zhu Y. Liu R. Zhang H. Wang Z.-s. Wu H. (2016). A Porphyrin Derivative for the Fabrication of Highly Stable and Sensitive Electrochemical Sensor and its Analytical Applications. Sensors Actuators B: Chem. 233, 206–213. 10.1016/j.snb.2016.04.049

[B12] Georgakilas V. Tiwari J. N. Kemp K. C. Perman J. A. Bourlinos A. B. Kim K. S. (2016). Noncovalent Functionalization of Graphene and Graphene Oxide for Energy Materials, Biosensing, Catalytic, and Biomedical Applications. Chem. Rev. 116 (9), 5464–5519. 10.1021/acs.chemrev.5b00620 27033639

[B13] Golsheikh A. M. Huang N. M. Lim H. N. Chia C. H. Harrison I. Muhamad M. R. (2013). One-pot Hydrothermal Synthesis and Characterization of FeS2 (Pyrite)/graphene Nanocomposite. Chem. Eng. J. 218, 276–284. 10.1016/j.cej.2012.09.082

[B14] Guo J. Yan L. Geng J. Zhu G. Han G.-Z. (2019). *In-Situ* Covalent Synthesis of Gold Nanorods on GO Surface as Ultrasensitive Raman Probe. Appl. Organometal Chem. 33 (4), e4791–4798. 10.1002/aoc.4791

[B15] Hsu S.-Y. Lee C.-L. Kuo C.-H. Kuo W.-C. (2021). Defective Graphene Nanosheets with Heteroatom Doping as Hydrogen Peroxide Reduction Catalysts and Sensors. Sensors Actuators B: Chem. 328, 129015–129024. 10.1016/j.snb.2020.129015

[B16] Hu H. Zhao Z. Wan W. Gogotsi Y. Qiu J. (2013). Ultralight and Highly Compressible Graphene Aerogels. Adv. Mater. 25 (15), 2219–2223. 10.1002/adma.201204530 23418081

[B17] Hu N. Meng L. Gao R. Wang Y. Chai J. Yang Z. (2011). A Facile Route for the Large Scale Fabrication of Graphene Oxide Papers and Their Mechanical Enhancement by Cross-Linking with Glutaraldehyde. Nano-micro Lett. 3, 215–222. 10.1007/BF03353675

[B18] Huang D. Li X. Chen M. Chen F. Wan Z. Rui R. (2019). An Electrochemical Sensor Based on a Porphyrin Dye-Functionalized Multi-Walled Carbon Nanotubes Hybrid for the Sensitive Determination of Ascorbic Acid. J. Electroanalytical Chem. 841, 101–106. 10.1016/j.jelechem.2019.04.041

[B19] Jiaojiao X. Pengyun W. Bin Z. Onyinye A. I. (2021). Enhancing Electrochemical Sensing for Catechol by Biomimetic Oxidase Covalently Functionalized Graphene Oxide. Bioproc. Biosyst Eng 44 (2), 343–353. 10.1007/s00449-020-02446-x 32968847

[B20] Li R. Yang Y. Wu D. Li K. Qin Y. Tao Y. (2019). Covalent Functionalization of Reduced Graphene Oxide Aerogels with Polyaniline for High Performance Supercapacitors. Chem. Commun. 55 (12), 1738–1741. 10.1039/c8cc07744d 30663746

[B21] Li X. Zheng W. Zhang L. Yu P. Lin Y. Su L. (2009). Effective Electrochemical Method for Investigation of Hemoglobin Unfolding Based on the Redox Property of Heme Groups at Glassy Carbon Electrodes. Anal. Chem. 81 (20), 8557–8563. 10.1021/ac9015215 19754140

[B22] Lin D. Wu J. Wang M. Yan F. Ju H. (2012). Triple Signal Amplification of Graphene Film, Polybead Carried Gold Nanoparticles as Tracing Tag and Silver Deposition for Ultrasensitive Electrochemical Immunosensing. Anal. Chem. 84 (8), 3662–3668. 10.1021/ac3001435 22439678

[B23] Liu H. Y. Ying T. L. Sun K. Qi D. Y. (1996). A Reagentless Biosensor Highly Sensitive to Hydrogen Peroxide Based on New Methylene Blue N Dispersed in Nafion” Gel as the Electron Shuttle. J. Electroanal. Chem. 417(1-2), 59–64. 10.1016/s0022-0728(96)04756-0

[B24] Liu M. An M. Xu J. Liu T. Wang L. Liu Y. (2021a). Three-dimensional Carbon Foam Supported NiO Nanosheets as Non-enzymatic Electrochemical H2O2 Sensors. Appl. Surf. Sci. 542, 148699. 10.1016/j.apsusc.2020.148699

[B25] Liu S. Cao S. Guo J. Luo L. Zhou Y. Lin C. (2018). Graphene Oxide-Silver Nanocomposites Modulate Biofilm Formation and Extracellular Polymeric Substance (EPS) Production. Nanoscale 10 (41), 19603–19611. 10.1039/C8NR04064H 30325394

[B26] Liu X. Zhang X. Zheng J. (2021b). One-pot Fabrication of AuNPs-Prussian Blue-Graphene Oxide Hybrid Nanomaterials for Non-enzymatic Hydrogen Peroxide Electrochemical Detection. Microchemical J. 160, 105595–105602. 10.1016/j.microc.2020.105595

[B27] Masibi K. K. Fayemi O. E. Adekunle A. S. Sherif E.-S. M. Ebenso E. E. (2018). Electrocatalysis of Lindane Using Antimony Oxide Nanoparticles Based-SWCNT/PANI Nanocomposites. Front. Chem. 6 (423), 423–438. 10.3389/fchem.2018.00423 30298128 PMC6160894

[B28] Mollarasouli F. Asadpour-Zeynali K. Campuzano S. Yáñez-Sedeño P. Pingarrón J. M. (2017). Non-enzymatic Hydrogen Peroxide Sensor Based on Graphene Quantum Dots-Chitosan/methylene Blue Hybrid Nanostructures. Electrochimica Acta 246, 303–314. 10.1016/j.electacta.2017.06.003

[B29] Nanda S. S. Papaefthymiou G. C. Yi D. K. (2015). Functionalization of Graphene Oxide and its Biomedical Applications. Crit. Rev. Solid State. Mater. Sci. 40 (5), 291–315. 10.1080/10408436.2014.1002604

[B30] Ovchinnikov O. V. Chernykh S. V. Smirnov M. S. Alpatova D. V. Vorob’eva R. P. Latyshev A. N. (2007). Analysis of Interaction between the Organic Dye Methylene Blue and the Surface of AgCl(I) Microcrystals. J. Appl. Spectrosc. 74 (6), 809–816. 10.1007/s10812-007-0126-4

[B31] Park M. Kim N. Lee J. Gu M. Kim B.-S. (2021). Versatile Graphene Oxide Nanosheets via Covalent Functionalization and Their Applications. Mater. Chem. Front. 5 (12), 4424–4444. 10.1039/d1qm00066g

[B32] Paul P. Mati S. S. Bhattacharya S. C. Kumar G. S. (2017). Spectroscopic, Calorimetric, Cyclic Voltammetric and Molecular Modeling Studies of New Methylene Blue-Polyadenylic Acid Interaction and Comparison to Thionine and Toluidine Blue O: Understanding Self-Structure Formation by Planar Dyes. Dyes Pigm. 136, 205–218. 10.1016/j.dyepig.2016.08.027

[B33] Paul P. Mati S. S. Kumar G. S. (2020). Insights on the Interaction of Phenothiazinium Dyes Methylene Blue and New Methylene Blue with Synthetic Duplex RNAs through Spectroscopy and Modeling. J. Photochem. Photobiol. B: Biol. 204, 111804. 10.1016/j.jphotobiol.2020.111804 32007677

[B34] Peña R. C. Bertotti M. Brett C. M. A. (2011). Methylene Blue/Multiwall Carbon Nanotube Modified Electrode for the Amperometric Determination of Hydrogen Peroxide. Electroanalysis 23 (10), 2290–2296. 10.1002/elan.201100324

[B35] Podsiadlo P. Kaushik A. K. Arruda E. M. Waas A. M. Shim B. S. Xu J. (2007). Ultrastrong and Stiff Layered Polymer Nanocomposites. Science 318 (5847), 80–83. 10.1126/science.1143176 17916728

[B36] Ramesh P. Bhagyalakshmi S. Sampath S. (2004). Preparation and Physicochemical and Electrochemical Characterization of Exfoliated Graphite Oxide. J. Colloid Interf. Sci. 274 (1), 95–102. 10.1016/j.jcis.2003.11.030 15120282

[B37] Sharma P. Hussain N. Borah D. J. Das M. R. (2013). Kinetics and Adsorption Behavior of the Methyl Blue at the Graphene Oxide/Reduced Graphene Oxide Nanosheet-Water Interface: A Comparative Study. J. Chem. Eng. Data 58 (12), 3477–3488. 10.1021/je400743r

[B38] Subbiah D. K. Nesakumar N. Kulandaisamy A. J. Rayappan J. B. B. (2017). Ferricyanide/reduced Graphene Oxide as Electron Mediator for the Electrochemical Detection of Methanol in Canned Citrus Sinensis and Citrus Limetta. Sensors Actuators B: Chem. 248, 708–717. 10.1016/j.snb.2017.03.168

[B39] Tuček J. Sofer Z. Bouša D. Pumera M. Holá K. Malá A. (2016). RETRACTED ARTICLE: Air-Stable Superparamagnetic Metal Nanoparticles Entrapped in Graphene Oxide Matrix. Nat. Commun. 7, 12879–12890. 10.1038/ncomms12879 27628898 PMC5027615

[B40] Wu H. Fan S. Jin X. Zhang H. Chen H. Dai Z. (2014a). Construction of a Zinc Porphyrin-Fullerene-Derivative Based Nonenzymatic Electrochemical Sensor for Sensitive Sensing of Hydrogen Peroxide and Nitrite. Anal. Chem. 86 (13), 6285–6290. 10.1021/ac500245k 24918264

[B41] Wu H. Fan S. Zhang W. Chen H. Peng L. Jin X. (2014b). Amperometric Immunosensor Based on Covalent Immobilization of New Methylene Blue and Penicillin Polyclonal Antibody for Determination of Penicillin G in Milk. Anal. Methods 6 (2), 497–502. 10.1039/c3ay41624k

[B42] Wu H. Li X. Chen M. Wang C. Wei T. Zhang H. (2018). A Nanohybrid Based on Porphyrin Dye Functionalized Graphene Oxide for the Application in Non-enzymatic Electrochemical Sensor. Electrochimica Acta 259, 355–364. 10.1016/j.electacta.2017.10.122

[B43] Xue Z. Fu X. Rao H. Zhou X. Liu X. Lu X. (2018). A New Electron Transfer Mediator Actuated Non-enzymatic Nitrite Sensor Based on the Voltammetry Synthetic Composites of 1-(2-Pyridylazo)-2-Naphthol Nanostructures Coated Electrochemical Reduced Graphene Oxide Nanosheets. Electrochimica Acta 260, 623–629. 10.1016/j.electacta.2017.11.181

[B44] Yao H. Li N. Xu S. Xu J. Zhu J. Chen H. (2005). Electrochemical Study of a New Methylene Blue/silicon Oxide Nanocomposition Mediator and its Application for Stable Biosensor of Hydrogen Peroxide. Biosens. Bioelectron. 21 (2), 372–377. 10.1016/j.bios.2004.08.051 16023965

[B45] Zhang D. Ouyang X. Li L. Dai B. Zhang Y. (2016). Real-time Amperometric Monitoring of Cellular Hydrogen Peroxide Based on Electrodeposited Reduced Graphene Oxide Incorporating Adsorption of Electroactive Methylene Blue Hybrid Composites. J. Electroanalytical Chem. 780, 60–67. 10.1016/j.jelechem.2016.09.005

