# Supplementary material for: New Methylene Blue Covalently Functionalized Graphene Oxide Nanocomposite as Interfacial Material for the Electroanalysis of Hydrogen Peroxide
Source: Front Chem. 2021 Dec 3;9:788804. doi: 10.3389/fchem.2021.788804 (PMC8677660; doi:10.3389/fchem.2021.788804)
Supplement: Supplementary file 1 [file DataSheet1.docx]

Supplementary Material for

New methylene blue covalently functionalized graphene oxide nanocomposite as interfacial material for the electroanalysis of hydrogen peroxide

**Jifang Chen^1^, Ziqing Gao^1^, Ruonan Yang^1^, Huiling Jiang^1^, Lin Bai ^1^, Ailong Shao ^1^*, Hai Wu^1,2^***

^1^School of Chemistry and Materials Engineering, Fuyang Normal University, Fuyang, China

^2^Anhui Province Key Laboratory of Environmental Hormone and Reproduction, Anhui Province Key Laboratory of Embryo Development and Reproductive Regulation, Fuyang, China

*** Correspondence:** Ailong Shao; Hai Wu

E-mail address: shaoailong2008@163.com; wuhai317@126.com.
